# Responses of *Acidobacteria* *Granulicella* sp. WH15 to High Carbon Revealed by Integrated Omics Analyses

**DOI:** 10.3390/microorganisms8020244

**Published:** 2020-02-12

**Authors:** Ohana Y.A. Costa, Marcelo M. Zerillo, Daniela Zühlke, Anna M. Kielak, Agata Pijl, Katharina Riedel, Eiko E. Kuramae

**Affiliations:** 1 Department of Microbial Ecology, Netherlands Institute of Ecology (NIOO-KNAW), Droevendaalsesteeg 10, 6708 PB Wageningen, The Netherlandsmarcelo.zerillo@gmail.com (M.M.Z.); a.kielak@nioo.knaw.nl (A.M.K.); a.pijl@nioo.knaw.nl (A.P.); 2 Institute of Microbiology, University of Greifswald, Felix-Hausdorff-Strasse 8, 17487 Greifswald, Germany; daniela.zuehlke@uni-greifswald.de (D.Z.); riedela@uni-greifswald.de (K.R.)

**Keywords:** genome, transcriptome, proteome, oligotroph, stress signal, transporters, sigma factor σW, cellobiose

## Abstract

The phylum *Acidobacteria* is widely distributed in soils, but few representatives have been cultured. In general, *Acidobacteria* are oligotrophs and exhibit slow growth under laboratory conditions. We sequenced the genome of *Granulicella* sp. WH15, a strain obtained from decaying wood, and determined the bacterial transcriptome and proteome under growth in poor medium with a low or high concentration of sugar. We detected the presence of 217 carbohydrate-associated enzymes in the genome of strain WH15. Integrated analysis of the transcriptomic and proteomic profiles showed that high sugar triggered a stress response. As part of this response, transcripts related to cell wall stress, such as sigma factor σW and toxin–antitoxin (TA) systems, were upregulated, as were several proteins involved in detoxification and repair, including MdtA and OprM. KEGG metabolic pathway analysis indicated the repression of carbon metabolism (especially the pentose phosphate pathway) and the reduction of protein synthesis, carbohydrate metabolism, and cell division, suggesting the arrest of cell activity and growth. In summary, the stress response of *Granulicella* sp. WH15 induced by the presence of a high sugar concentration in the medium resulted in the intensification of secretion functions to eliminate toxic compounds and the reallocation of resources to cell maintenance instead of growth.

## 1. Introduction

*Acidobacteria* is one of the most abundant bacterial phyla in soil, yet little is known about its physiology, ecological function, and impact on the soil environment [1]. The first species of *Acidobacteria* was described in the 1990s [2], and the ubiquity of the group was only recognized after the introduction of bacterial identification techniques that do not rely on bacterial isolation, such as 16S rRNA gene sequencing and metagenomics [1]. This phylum constitutes 20–50% of the soil bacterial community [1,3,4,5,6,7], but the few species that have been isolated exhibit slow growth under standard laboratory conditions, resulting in a relatively small number of cultured representatives. Genome analyses have revealed only one or two copies of the 16S rRNA gene in species sequenced to date [8,9,10], which may also indicate slow growth of these bacteria under natural conditions [11]. Consequently, the factor(s) responsible for the prevalence and successful adaptation of *Acidobacteria* to soil conditions remain unclear.

A strong negative correlation between *Acidobacteria* abundance based on 16S rRNA amplicon next-generation sequences and soil organic carbon content has been observed in diverse microbiome studies, leading to the conclusion that the phylum is composed of oligotrophic bacteria [12,13]. Oligotrophs are mainly characterized by their capacity to grow under low nutrient availability and their higher substrate utilization efficiency. In general, they are able to thrive in poor nutrient environments and exhibit slow growth under laboratory conditions [14]. Although most acidobacterial isolates have been obtained from low-nutrient culture media, some isolates are capable of growing in higher sugar concentrations, including strains from the genera *Granulicella*, *Edaphobacter* and strains similar to *Acidobacterium* [1,15,16].

Many soil *Acidobacteria* are able to degrade a wide range of carbon sources, mainly mono- and disaccharides such as glucose, xylose, mannose, galactose, and cellobiose. Predictions of genes associated with the degradation of polysaccharides in acidobacterial genomes have not always been confirmed by experimental data [1]. This gap highlights the need for studies based on cultured strains. We recently established a culture medium and incubation conditions permitting larger amounts of acidobacterial biomass (cells and/or exopolysaccharides, EPSs) to be harvested after 4 days of incubation [15]. By contrast, on other reported media, most cultivated acidobacterial species form visible colonies only after weeks of incubation [17]. As no study has addressed the response of acidobacterial strains under different sugar concentrations, the aim of this study was to sequence the genome of an acidobacterial strain, *Granulicella* sp. WH15, and determine the transcriptome and proteome responses under conditions of low (0.025%) and high (3%) sugar cellobiose concentrations.

## 2. Materials and Methods

### 2.1. Genome

The *Granulicella* sp. WH15 strain [18] obtained from the collection of the Netherlands Institute of Ecology (NIOO-KNAW) was grown on 1/10 TSB agar medium [18] at pH 5.0 for 3 days at 30 °C. The bacterial cells were harvested and the genomic DNA was extracted using a MasterPure™ DNA Purification Kit (Epicentre, Madison, WI, USA) according to the manufacturer’s instructions. A total of 10 µg of DNA was sent to the Genomics Resource Center (Baltimore, MD, USA) for a single long insert library (15–20 kb), which was constructed and sequenced in one SMRTcell using the PacBio RS II (Pacific Biosciences, Inc., CA, USA) sequencing platform. De novo assembly was performed with the help of SMRT Analysis software v2.2.0 (Pacific Biosciences, Inc., CA, USA) featuring HGAP 2 [19] and subsequent correction with Pilon 1.16 [20] to reveal a circular replicon: a 4,675,153-bp chromosome (G+C content 60.7%; 58.4× coverage). Automatic gene prediction and annotation was performed using Prokka [21], and RAST genome annotation server (http://rast.nmpdr.org/) [22]. Genes were mapped to COG (Cluster of Orthologous Groups) and KEGG IDs using the COG database (2014 release) [23] and KEGG database (release 2013) [24], using in-house scripts. The CAZyme contents of WH15 genome were determined by identifying genes containing CAZyme domains using the dbCAN2 meta server (http://bcb.unl.edu/dbCAN2/) [25], according to the CAZy (Carbohydrate-Active Enzyme) database classification [26]. Only CAZyme domains predicted by at least two of the three algorithms (DIAMOND, HMMER, and Hotpep) employed by dbCAN2 were kept. A circular genome map was drawn used CGView software [27]. Average nucleotide identity (ANI) between strain WH15 and other available *Granulicella* genomes was calculated using the webtool ANI calculator, available at https://www.ezbiocloud.net/tools/ani [28].

### 2.2. Growth Experiments

CAZyme annotation was compared to in vitro carbohydrate utilization assays. Modified 1/10 TSB (1.7 g of casein hydrated (acid) (Oxoid^TM^_,_ Basingstoke, UK), 0.3 g of tryptone (vegetable) (Fluka^TM^ 95039, Seelze, Germany), 0.5 g of sodium chloride, and 0.25 g of dipotassium phosphate (Sigma-Aldrich^®^, St. Louis, MO, USA) in 1 liter of distilled water) pH 5.0 medium was supplemented with single carbon sources: pectin (apple; Sigma-Aldrich), glycogen (Sigma-Aldrich), glucosamine (Sigma-Aldrich), and cellulose (Sigma-Aldrich), at 1% (*w/v*), and D-glucose, D-galactose, D-mannose, D-xylose, L- arabinose, L-rhamnose, D-galacturonic acid, cellobiose, D-lactose, and sucrose at 25 mM. Plates were inoculated with 100 μL of cell suspension at OD_600nm_ = 1 obtained from modified TSB (3% of cellobiose), homogenized, and incubated at 30 °C for 72 h, at which point growth was evaluated. After 72 h of growth, colonies were reinoculated in a new plate with the same carbohydrate concentration, and the process was repeated three times in order to confirm growth.

### 2.3. Transcriptome

Bacteria were grown in two sugar concentrations, based on solid (1.5% agarose) modified 1/10 TSB medium pH 5.0 (as described above) supplemented with 0.025% and 3% of cellobiose for low- and high sugar conditions, respectively. Plates were inoculated with 100 μL of cells at OD_600nm_ = 1 nm/mL, homogenized, and incubated at 30 °C for 72 h, when the strain produces colonies in culture medium and enough bacterial biomass for material extraction [15]. Each treatment had three replicates, represented by individual plates.

RNA extraction was performed using Aurum Total RNA kit (Bio-Rad^®^, Hercules, CA, USA) in a final volume of 50 µL of elution buffer. A total of 6 µg of RNA was rRNA depleted with the Ribo-Zero Bacteria Kit from Illumina. The sequencing library of each sample was prepared with the Kapa Biosystems Stranded RNA-Seq kit, and sequenced according to the Illumina TruSeq v3 protocol on the HiSeq2000 with a single read 50 bp and 7 bp index at Erasmus Center for Biomics (Rotterdam, NL). The quality of the raw fastq files was checked and filtered with FastQC. Read alignment was performed on the genome of WH15 using Bowtie2 [29]. Differential expression profiling was performed between low and high sugar treatments. Gene expression levels were quantified using the software package RNASeq by Expectation Maximization (RSEM) [30]. The matrix of fragment counts from each sample was used for the differential expression analysis via the edgeR package [31]. Differentially expressed genes between the two treatments, using the low sugar treatment as a reference, were identified at a significance level of 0.05 with a false discovery rate (FDR) correction method and the log_2_ fold change (logFC) equaled to 1. To generate a heatmap, 1% differentially expressed genes were selected. COG [23] and KEGG [24] analyses were performed for differentially expressed genes. 

### 2.4. Analysis of the Cytosolic Proteome by Mass Spectrometry and Data Analysis

Bacteria were grown in the same low sugar (0.025% cellobiose, LS) and high sugar (3% of cellobiose, HS) conditions for transcriptome analyses. Similarly, each treatment had three replicates, represented by individual plates. Bacterial biomass from each low and high sugar treatment were collected from plates and resuspended in 1 mL TE buffer. Bacterial cells were harvested by centrifugation 10,015× *g* at 4 °C for 10 min. Pellets were washed twice with 1 mL of TE buffer and finally resuspended in 1 mL TE buffer. Then, 500µL of cell suspension was transferred into 2 mL screw cap tubes filled with 500 µL glass beads (0.1 mm in diameter; Sarstedt, Nümbrecht, Germany) and mechanically disrupted using Fastprep (MP Biomedicals, Irvine, CA, USA) for 3 × 30 s at 6.5 m/s, with on-ice incubation for 5 min between cycles. To remove cell debris and glass beads, samples were centrifuged for 10 min at 4 °C at 21,885× *g*, followed by a second centrifugation (30 min at 4 °C at 21,885× *g*) to remove insoluble and aggregated proteins. The protein extracts were kept at −20 °C. Protein concentration was determined using RotiNanoquant (Carl Roth, Karlsruhe, Germany). Proteins were separated by SDS-PAGE. Protein lanes were cut into ten equidistant pieces and in-gel digested using trypsin as described earlier [32]. Tryptic peptides were separated on an EASY-nLC II coupled to an LTQ Orbitrap Velos using a non-linear binary 76 min gradient from 5–75% buffer B (0.1% acetic acid in acetonitrile) at a flow rate of 300 nL/min and infused into an LTQ Orbitrap Velos (Thermo Fisher Scientific, Waltham, MA, USA) mass spectrometer. Survey scans were recorded in the Orbitrap at a resolution of 60,000 in the *m/z* range of 300–1700. The 20 most intense peaks were selected for collision-induced dissociation (CID) fragmentation in the LTQ. Dynamic exclusion of precursor ions was set to 20 s; single-charged ions and ions with unknown charge were excluded from fragmentation; internal calibration was applied (lock mass 445.120025). 

For protein identification, resulting MS/MS spectra were searched against a database containing protein sequences of *Granulicella* sp. strain WH15 and common laboratory contaminants (9236 entries) using Sorcerer-Sequest v.27, rev. 11 (Thermo Fisher Scientific, Waltham, MA, USA) and Scaffold v4.8.4 (Proteome Software, Portland, OR, USA) as described earlier [33]. Relative quantification of proteins was based on normalized spectrum abundance factors (NSAFs; [34]). Statistical analysis was done using MeV [35]; *t*-test was applied for proteins that were identified in at least two replicates of the respective condition. Hierarchical clustering and *t*-test of z-transformed normalized data were performed with the following parameters: unequal group variances were assumed (Welch approximation), *p*-values based on all permutation with *p* = 0.01, significance determined by adjusted Bonferroni correction. Only significantly changed proteins showing at least two-fold changes between conditions were considered for further analysis. Furthermore, so-called on/off proteins that were only identified in one condition were analyzed. Functional classification of *Granulicella* sp. strain WH15 proteins was carried out using Prophane software (www.prophane.de; [36]), COG [23], and KEGG databases [24]. Voronoi treemaps were generated with Paver software (Decodon GmbH, Greifswald, Germany). 

### 2.5. Data Availability

The *Granulicella* sp. WH15 strain genome data are deposited at NCBI with accession number CP042596. Transcriptome data are deposited in the NCBI GEO database with accession number GSM4017160-65. The mass spectrometry proteomics data are deposited to the ProteomeXchange Consortium via the PRIDE [37] partner repository with the dataset identifier PXD015715.

## 3. Results

### 3.1. Genome Annotation and CAZymes

**Figure 1:**
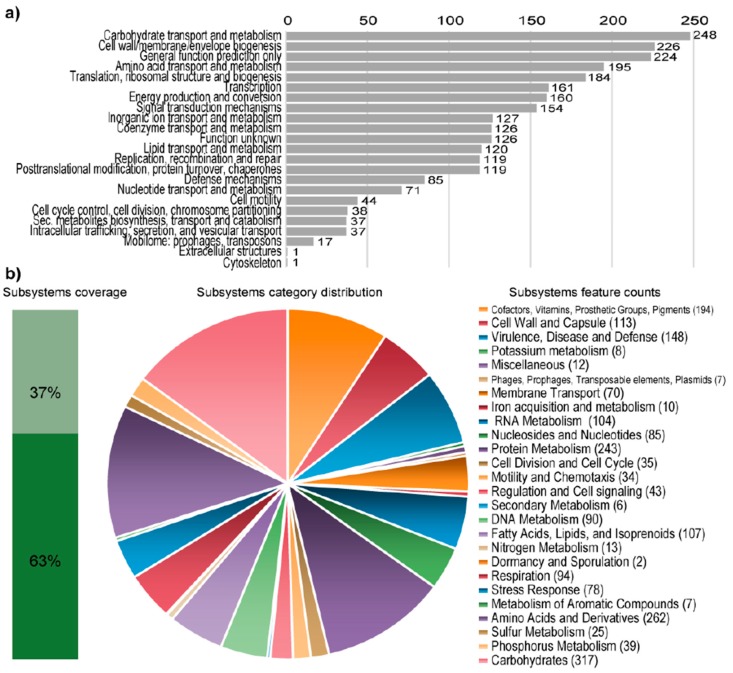
Statistics of the COG and RAST subsystem annotations of *Granulicella* sp. WH15. (**a**) COG (Cluster of Orthologous Groups) category distribution showing the number of genes annotated in each category. (**b**) Subsystem category distribution. The light-green bar represents the percentage of proteins that could be annotated by the RAST Server, and the dark-green bar represents the proteins that were not annotated. The pie chart represents the number of proteins annotated to each subsystem category.

**Figure 2:**
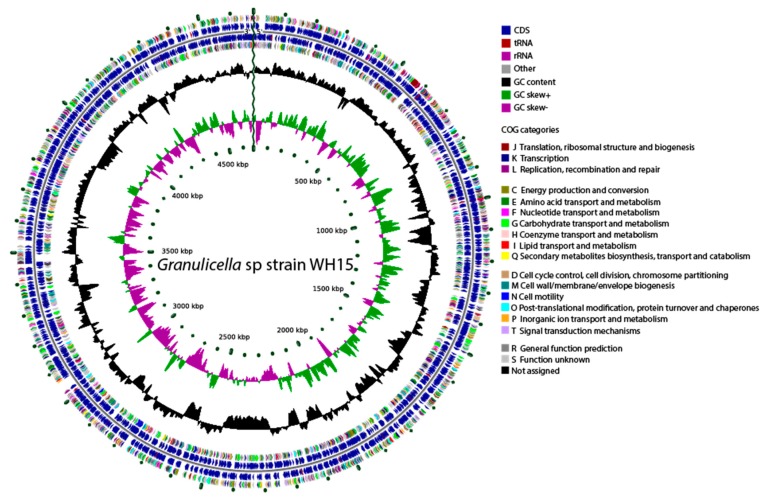
Graphical circular genome map of *Granulicella* sp. WH15. The rings indicate coding sequences, COG categories, GC content, and GC skew.

The assembled genome of *Granulicella* sp. WH15 is 4,673,153 bp, with 60.7% GC content, 3849 proteins, and only one rRNA operon. Functional annotation using COG (Cluster of Orthologous Groups) and RAST analysis resulted in the classification of 2620 genes into 23 COG functional groups and the annotation of 1456 genes to RAST subsystems. The properties and distribution of genes into COGs/RAST functional categories are listed in Table 1 and Figure 1, respectively. A circular genome map of WH15 is depicted in Figure 2.

RAST analysis showed that only 37% (1456/3939) of the annotated genes were assigned to subsystems. Among the subsystem categories present in the genome, carbohydrates and dormancy and sporulation had the highest and lowest feature counts, respectively (Figure 1). A comparison with five publicly available genomes of *Granulicella* strains showed that the genome of strain WH15 is larger only than that of G. *pectinivorans* (Table 2). The average nucleotide identity (ANI) values (Table 2) showed that *Granulicella* strain WH15 strain does not belong to any of the species for which the genomes have been sequenced.

We also performed automatic annotation followed by manual curation of CAZymes by using a dbCAN2 search, which revealed 217 CAZyme genes in the strain WH15 genome (E-value < 10^−5^) (Table 3). This value is similar to that of other soil *Acidobacteria* Gp1 species determined using the same parameters [38].

Our annotation of strain WH15’s genome revealed only four genes involved in pectin degradation. Based on the presence of polygalacturonase genes (GH28 family: GWH15_00665; GWH15_06350; GWH15_16190; GWH15_17915), strain WH15 might be capable of breaching the complex heteropolysaccharides of pectins, but lacks key genes for breaking the pectin backbone (PL1 and PL4), genes targeting D-xylose substitutions and side chains (GH53, GH54, GH93, and GH127), or genes for further pectin saccharification (GH88 and GH105). Accordingly, we did not observe growth of strain WH15 on media supplemented with pectin or galacturonic acid, in contrast to most sequenced soil isolates of *Acidobacteria* (especially *Granulicella*) [39,40,41]. However, growth was obtained on media containing sucrose, glucose, cellobiose, xylose, arabinose, mannose, rhamnose, galactose, or lactose (Supplementary Table S1). These results are consistent with the presence of α-L-rhamnosidase (GWH15_06355; GWH15_03775) and β-galactosidase (GWH15_05655, GWH15_06300; GWH15_07745) genes in the strain WH15 genome. In addition, the strain WH15 genome contained *acsC* (GWH15_12725), *acsAB* (GWH15_03685), and *bcsC* (GWH15_13465) genes involved in cellulose biosynthesis.

Analysis with the ANTISMASH 4.2.0 database revealed the presence of 33 biosynthetic gene clusters, including 11 of defined type (Table 4) and 22 classified as putative. Among the putative clusters, two have similarities with known clusters involved in the production of O-antigen and thuggacin (Table 4). In addition, other identified clusters showed potential for the production of terpenes, bacteriocins, polyketide synthases (PKSs), fatty acids, lasso peptides, and saccharides.

### 3.2. Transcriptome Analysis

**Figure 3:**
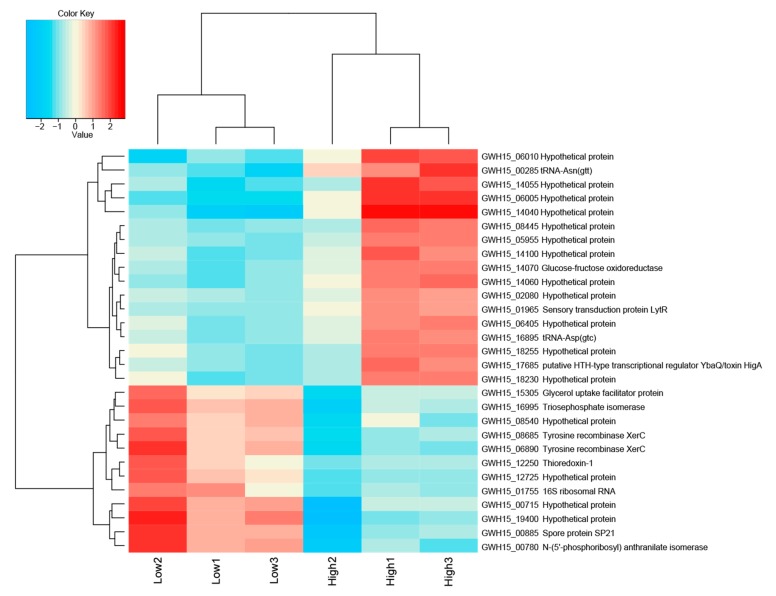
Heatmap of transcriptome data showing the top differentially expressed genes log_2_ > 1.5-fold and hierarchical clustering analysis in high-sugar versus low-sugar conditions. High to low expression is shown by a gradient color from red to blue, respectively.

The total numbers of reads in the low and high sugar conditions are listed in Supplementary Table S2. In total, 106 (53 upregulated and 53 downregulated) genes were significantly differentially expressed (*p*-value > 0.05) in both treatments, of which only 44 could be annotated, reflecting current gaps in the knowledge of the genomes of *Acidobacteria* in general. Gene expression analysis of *Granulicella* sp. WH15 grown in low and high sugar conditions showed that 28 genes were upregulated at log_2_ > 1.0-fold in the high sugar treatment, of which 17 were induced at log_2_ > 1.5-fold (Supplementary Table S3). In addition, 30 genes were downregulated at log_2_ > 1.0-fold in the high-sugar treatment, of which 12 were repressed at log_2_ > 1.5-fold (Figure 3) (Supplementary Table S3). 

**Figure 4:**
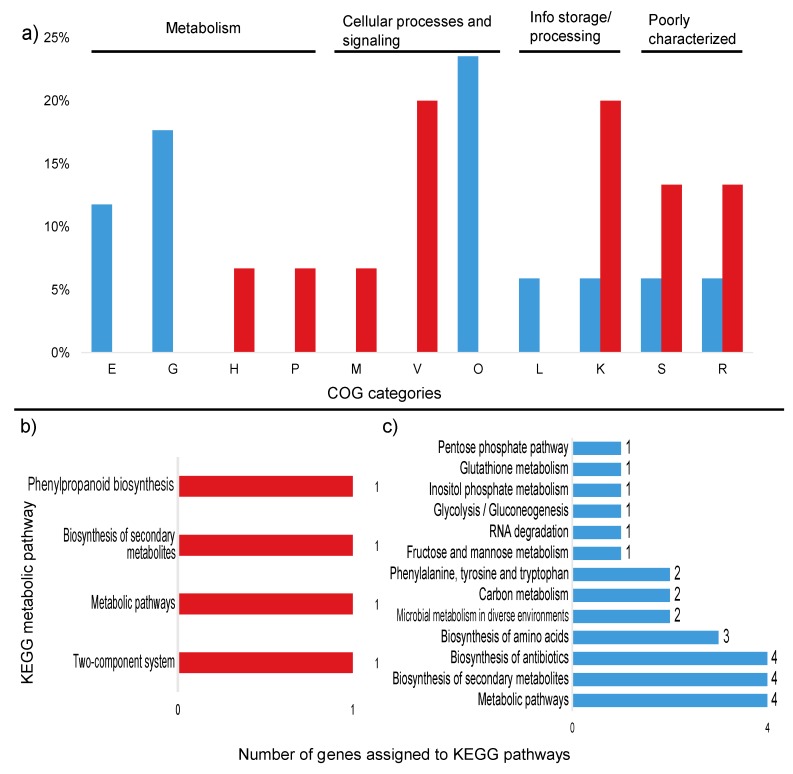
Percentage of differentially expressed transcripts assigned to COG categories and number of KEGG pathways up and down regulated in the high sugar treatment. (**a**) Percentage of upregulated (red) and downregulated (blue) transcripts assigned to COG categories. (**b**) Number of upregulated transcripts assigned to KEGG pathways. (**c**) Number of downregulated transcripts assigned to KEGG pathways. Unassigned transcripts are not shown. E: amino acid transport and metabolism; G: carbohydrate transport and metabolism; H: coenzyme transport and metabolism; M: cell wall/membrane/envelope biogenesis; V: defense mechanisms; P: inorganic ion transport and metabolism; O: post-translational modification; L: replication, recombination, and repair; K: transcription; S: function unknown; R: general function and prediction.

The comparison between the treatments demonstrated that the high sugar condition mainly induced the expression of genes related to stress, as well as several unknown hypothetical proteins. Among the annotated genes, a transfer RNA (tRNA-Asn-GTT) was the most upregulated (2.7-fold). In addition, the expression of the genes *gfo4* (glucose-fructose oxidase), *tRNA-Asp* (GTC), *higA* (HigA antitoxin), and *lytR* (sensory transduction protein LytR) was upregulated (>1.5-fold) in the high-sugar condition, and the expression of genes coding for the stress response sigma factor SigW (σW), peroxiredoxin TsaA, and toxin HigB-1 was upregulated >1.0-fold. By contrast, the high-sugar condition suppressed the expression of genes related to cell cycle/division and energy metabolism. Downregulation of expression (<1.5-fold) was observed for the genes *trpF* (N-(5’-phosphoribosyl) anthranilate isomerase) and *ssrA* (transfer-messenger RNA), which are involved in amino acid/protein synthesis; two copies of *xerC* (tyrosine recombinase XerC) and the 16S ribosomal RNA gene, which are involved in cell division; *tpiA* (triosephosphate isomerase) and *glpF* (glycerol uptake facilitator protein), which are involved in carbohydrate transport/metabolism; and *hspA* (spore protein SP21), a heat shock chaperone. The expression of genes encoding the housekeeping sigma factor RpoD, 6-phosphogluconate dehydrogenase (*gndA*), RNase P, and Lon protease 2 (*lon2*) was also significantly downregulated (>1.0-fold). COG category assignments of the differentially expressed transcripts are presented in Figure 4a. All of the significantly up- and downregulated genes (>1.0 fold) and their annotations are described in Supplementary Table S3. 

Among the upregulated transcripts, two genes could be assigned to KEGG pathways: *tsaA* (K11188—metabolic pathways, biosynthesis of secondary metabolites and phenylpropanoid biosynthesis) and *lytR* (K07705—two-component system) (Figure 4a). Of the downregulated transcripts, five genes were linked to metabolic pathways in the KEGG database: *trpF* (5 pathways), *tpiA* (10 pathways), *dnaK* (1 pathway), *trpC* (5 pathways), and *gndA* (8 pathways) (Figure 4b). These genes are mostly related to the production of antibiotics and secondary metabolites, as well as amino acid and carbon metabolism. The metabolic pathways for each gene are described in Table 5.

### 3.3. Proteome Analysis

**Figure 5:**
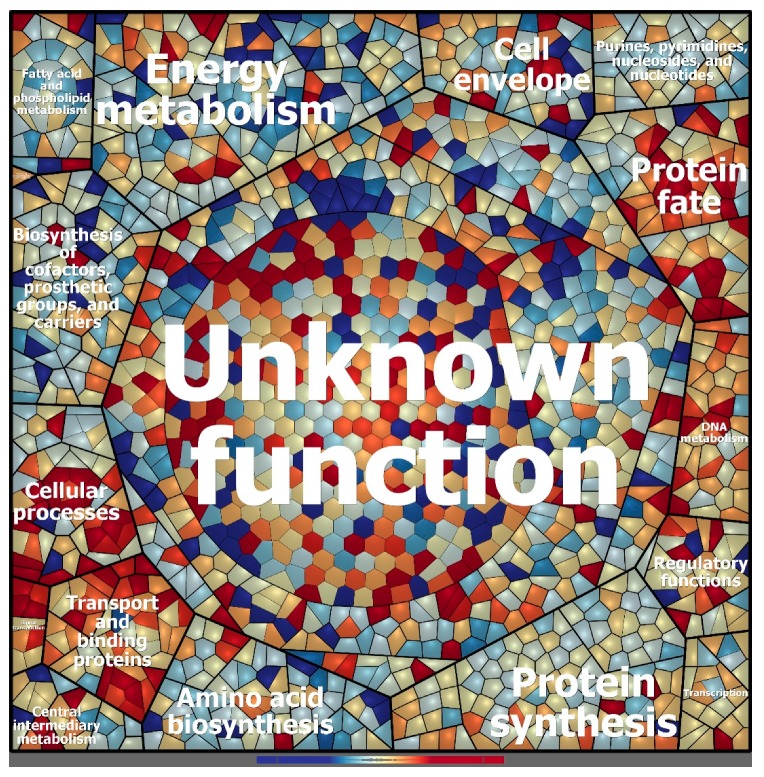
Voronoi treemap visualization of the protein expression patterns of *Granulicella* sp. WH15 under low and high sugar conditions. Functional classification was conducted using Prophane 2.0 (www.prophane.de) and is based on TIGRFAMs, where the function level indicates “main role” and the B function level indicates “subrole”. Each cell represents a quantified protein; proteins are clustered according to their function. Proteins expressed in higher amounts under low sugar conditions are depicted in blue, and proteins expressed in higher amounts under high sugar conditions are depicted in red.

**Figure 6:**
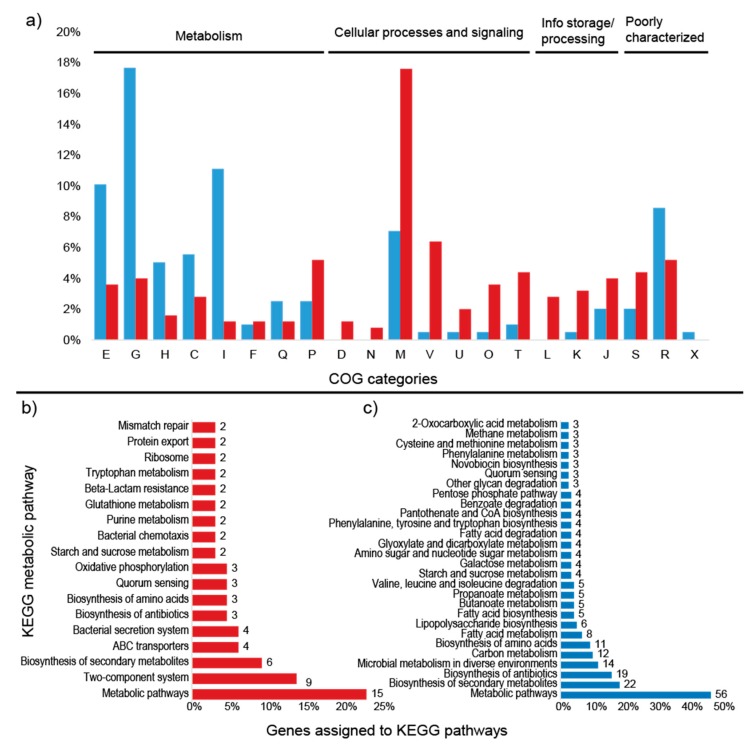
Percentage of differentially expressed proteins assigned to COG categories and number of KEGG pathways. (**a**) Percentage of upregulated (red) and downregulated (blue) proteins assigned to COG categories; (**b**) number of upregulated proteins assigned to KEGG pathways; (**c**) number of downregulated proteins assigned to KEGG pathways. Unassigned proteins are not shown. E: amino acid transport and metabolism; G: carbohydrate transport and metabolism; H: coenzyme transport and metabolism; C: energy production and conversion; I: lipid transport and metabolism; F: nucleotide transport and metabolism; Q: secondary metabolites; D: cell cycle; N: cell motility; M: cell wall/membrane/envelope biogenesis; V: defense mechanisms; P: inorganic ion transport and metabolism; U: intracellular trafficking; O: post-translational modification; T: signal transduction mechanisms; L: replication, recombination, and repair; K: transcription; J: translation; S: function unknown; R: general function and prediction; X: mobilome.

Qualitative analysis of the proteomic data demonstrated that the samples clustered according to treatment (i.e., high or low sugar condition; Supplementary Figure S1). In total, 1,418 proteins could be identified in both conditions in two of three replicates each. Overall, 448 proteins showed significant differences between the low and high sugar conditions (*t*-test, *p* = 0.01; Supplementary Figure S2). In addition, 249 so-called on/off proteins that were only present in one condition were detected. The proteome patterns of WH15 under the low and high sugar treatments are depicted in Figure 5. In the high sugar condition, 121 proteins were upregulated two-fold, and 129 proteins were “on”; 78 proteins were downregulated at least two-fold, and 120 proteins were “off” (Figure 5; Supplementary Tables S4 and S5). A comparison with the transcriptome data revealed that two genes (*lytR* and a hypothetical protein—ORF 05985) were upregulated and two genes (*gndA* and a hypothetical protein—ORF 08600) were downregulated in both datasets for the high sugar treatment. Among the differentially expressed proteins, 332 could be assigned to COG categories, and 184 were annotated to KEGG orthologs (Figure 6).

### 3.4. Upregulated Proteins

**Figure 7:**
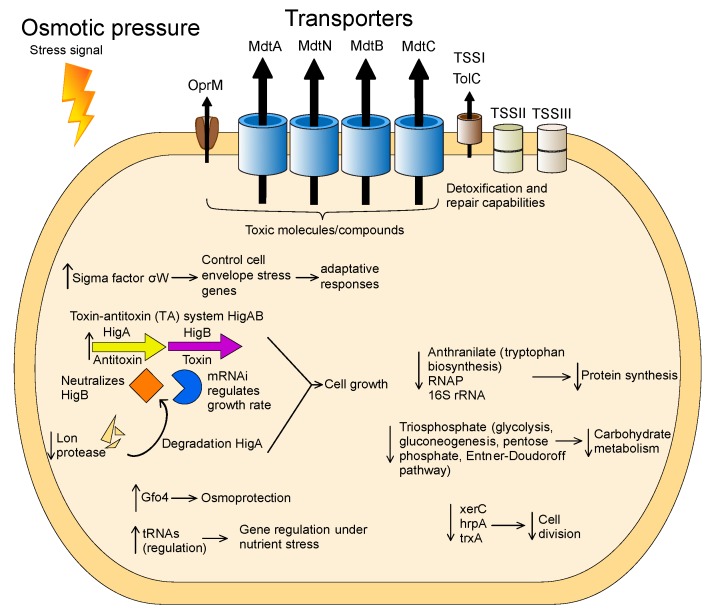
Model of the cellular processes involved in osmotic stress adaptation in *Granulicella* sp. WH15. The arrows depict upregulation (↑) and downregulation (↓) of transcripts and proteins.

COG analysis demonstrated that most of the upregulated proteins belong to the cell wall/membrane/envelope biogenesis (44) and defense mechanism (16) categories (Figure 6a). TigrFam classification grouped most of the upregulated/on proteins in the protein fate category (27), followed by the cellular processes (21), transport/binding proteins (20), and cell wall/membrane/envelope biogenesis categories (12) (Supplementary Figure S3). Closer examination of the subroles of the most abundant protein categories demonstrated that their main functions were related to peptide secretion and trafficking, detoxification and toxin production/resistance processes, efflux pumps, transporters, and TonB-dependent receptors (Supplementary Table S4). In addition to the sensory transduction protein LytR (*lytR*), which was also upregulated in the transcriptome data, several cell membrane proteins could be identified, such as the outer membrane protein OprM (*oprM* 1, 4, 5, 6, and 7), the LPS-assembly protein LptD (*lptD* 2), the macrolide export proteins MacA and MacB (*macA* 1, *macB* 6 and 8), the polysialic acid transport protein KpsD (*kpsD* 2), and the multidrug resistance proteins MdtB and MdtC (*mdtB* 2 and 3, *mdtC* 3 and 6). Among the upregulated proteins, 135 were annotated to KEGG orthologs, but only 44 orthologs could be mapped to KEGG metabolic pathways (Figure 6b, Supplementary Table S5), and some identifiers were assigned to more than one pathway. Most of the proteins were mapped to “general” metabolic pathways (15), two-component systems (9), biosynthesis of secondary metabolites (6), ABC transporters (4), and bacterial secretion systems (4), indicating that no particular metabolic pathway seemed to be specifically stimulated under the high sugar condition (Supplementary Figure S4), although the expression of many membrane proteins was enhanced (Figure 7). However, when the genes assigned to the COG carbohydrate transport and metabolism category were examined, proteins related to trehalose metabolism were observed, such as OtsA (trehalose-6-phosphate synthase), TreS (trehalose synthase) and GlgE 1 (alpha-1,4-glucan:maltose-1-phosphate maltosyltransferase), as well as a hypothetical protein similar to cellobiose phosphorylase (ORF GWH15_11910). Another interesting protein identified was CcpA (catabolite control protein A), a carbon metabolism regulator (Supplementary Table S4).

### 3.5. Downregulated Proteins

Within the COG categories, the downregulated proteins were mostly distributed among the carbohydrate transport and metabolism (35), amino acid transport and metabolism (20), and lipid transport and metabolism (22) categories (Figure 6a). TigrFam classified most of the downregulated/off proteins in the categories of energy metabolism (29) and biosynthesis of cofactors, prosthetic groups and carriers (13) (Supplementary Figure S3). Among the TigrFam subroles of the most abundant categories, most of the proteins were related to sugar metabolism and the biosynthesis of vitamins. In total, 119 proteins were assigned to KEGG orthologs, and 68 orthologs were assigned to KEGG metabolic pathways. The majority of the proteins were assigned to “general” metabolic pathways (56), biosynthesis of secondary metabolites (22), biosynthesis of antibiotics (19), microbial metabolism in diverse environments (12), carbon metabolism (12), and biosynthesis of amino acids (11) (Figure 6b). Consistent with the COG categories, a downregulation of proteins linked to KEGG pathways involved in carbon, amino acid, and lipid metabolism was observed (Supplementary Figure S4, Supplementary Table S6). In addition to general carbon metabolism, pathways related to secondary carbon sources seemed to be repressed, such as the metabolism of starch, sucrose, galactose, amino sugars, nucleotide sugars, fructose, mannose and other glycans, the pentose phosphate pathway, and glycolysis (Figure 6b, Figure 7, Supplementary Table S5). A repression of pathways related to several amino acids (e.g., valine, leucine, phenylalanine, tyrosine, and tryptophan) and the metabolism of fatty acids and lipopolysaccharides was observed (Figure 6b). The carbon-related repressed proteins included beta galactosidases (BgaA and BgaB), 1,4-beta-D-glucan glucohydrolase (GghA), xylan 1,4-beta-xylosidase (Xyl3A 1), beta-xylosidase (XynB), endopolygalacturonase (PehA 1), and exo-poly-alpha-D-galacturonosidase (PehX). Furthermore, four enzymes involved in the pentose phosphate pathway were downregulated: 6-phosphogluconate dehydrogenase, NADP(+)-dependent decarboxylase (GndA), transketolase 2 (TktB 1), gluconolactonase (Gnl 1), and KHG/KDPG aldolase (Eda). Among the proteins related to amino acid metabolism, the glutamate-pyruvate aminotransferase AlaA (alanine), histidinol-phosphate aminotransferase HisC2 (histidine), and prephenate dehydrogenase TyrA2 (tyrosine) were repressed. Enzymes involved in fatty acid degradation such as 3-ketoacyl-CoA thiolase (FadA), acyl-CoA dehydrogenase (FadE), and 3-hydroxyacyl-CoA dehydrogenase (FadN) were also downregulated (Supplementary Table S6).

## 4. Discussion

*Granulicella* sp. WH15, an isolate collected from soil containing decaying wood, has a genome consisting of 4.7 Mbp. Similar to other *Granulicella* strains, the genomic data suggest that *Granulicella* sp. WH15 is involved in the carbon cycling process in soil as well as the hydrolysis and utilization of stored carbohydrates and biosynthesis of exopolymers [40,41]. The saccharide gene clusters revealed by ANTISMASH analysis are likely involved in the production of capsule, antigen, and exopolymers. Exopolymer production by this strain has been observed under laboratory conditions, and the composition of the biopolymer has been characterized [42]. Members of the phylum *Acidobacteria* are generally considered slow-growing organisms that succeed in oligotrophic environments. The acidobacterium *Granulicella* sp. was isolated by employing a low-nutrient culture medium [18], similar to other strains of *Granulicella* [9,39]. Although this strain can be cultivated using low quantities of nutrients, it also develops well in higher concentrations of sugar [15]. To better understand the behavior of *Granulicella* sp. WH15 in response to different carbon source concentrations, comparative transcriptomic and proteomic analyses were performed under 0.025% and 3% cellobiose. The comparative transcriptomic and proteomic profiles of strain WH15 under low and high sugar conditions demonstrated that the higher concentration of carbon source triggered a stress response. Stress conditions in bacteria induce changes at the transcriptional level that are often associated with a variety of σ factors, which bind to RNA polymerase. Transcriptomic profiling showed that, in addition to other stress proteins, the sigma factor σW was upregulated log_2_ > 1.0-fold. In *Bacillus subtilis*, the σW regulon controls genes related to cell-envelope stress, membrane proteins, and proteins involved in protection against toxins or antibiotics. Expression of this regulon has been observed under conditions of stress that affect cell wall biosynthesis or membrane integrity, such as alkali shock, salt stress, and treatments with cationic peptides and detergents [43]. The cell envelope is the most external form of bacterial defense and receives stress stimuli, senses perturbations, and transmits signals that induce the transcriptional changes necessary for an adaptive response [44]. Another stress protein upregulated in the high sugar condition was the toxin–antitoxin (TA) system HigAB. The prokaryotic TA system encodes a stable toxin (HigB) and an unstable antitoxin (HigA) that neutralizes HigB. HigB is an mRNA interferase that is believed to be involved in growth rate control [45]. Expression of the system is induced by various stress stimuli, such as nutritional stress, heat shock [46], and exposure to chloramphenicol and chloroform [47,48]. In *Escherichia coli*, amino acid starvation strongly induces the transcription of the TA system [45]. The higher expression level of the antitoxin HigA compared to HigB in this study suggests that the acidobacterial cells were adapting to stress [49] and therefore produced more antitoxin to counterbalance the effect of HigB. In addition, these results are compatible with the downregulation of Lon protease, which contributes to the proteolytic regulation of many cell functions and is involved in the degradation of HigA during amino acid starvation [50]. The upregulated gene *gfo4* encodes glucose-fructose oxidoreductase—an enzyme involved in the production of molecules that function in osmoprotection under high sugar conditions [51]. The production of this enzyme could be important to counterbalance the effects of the high amounts of sugar in the culture medium. By contrast, the gene *glpF*, which encodes a glycerol uptake facilitator protein, was downregulated. This aquaglyceroporin facilitates the transport of glycerol and other linear polyalcohols across membranes, which would be redundant, as the cell is able to produce such molecules via the glucose-fructose oxidoreductase gfo4. 

Another upregulated protein, LytR, is a sensory transduction protein that is part of the LytSR system, which in *Staphylococcus aureus* functions as a sensor-response system that detects perturbations in the electrical potential of the cell membrane, such as disturbances caused by the presence of stress agents [52]. Furthermore, the upregulation of tRNA genes could be involved in expression regulation. In addition to their central role in protein synthesis, tRNAs are involved in the regulation of global gene expression during nutritional stresses to adapt microbial metabolism to changes in amino acid concentrations [53]. In the yeast *Saccharomyces cerevisiae*, for instance, glutamine tRNA is responsible for signaling nitrogen source quality in the environment and acts as a development regulator [54]. In addition, under different stress conditions, the relative abundances of tRNAs in *S. cerevisiae* change towards tRNAs recognizing rare codons to induce faster translation of the stress proteins necessary for adaptation [55]. Although these functions of tRNAs were described in yeast, similar functions may be present in bacteria such as strain WH15. 

The proteomic profile of the high sugar treatment was consistent with the upregulation of stress-related genes observed in the transcriptomic analyses. Most of the upregulated/on proteins were membrane proteins related to peptide secretion and trafficking, detoxification and toxin resistance, efflux pumps and transporters, and cell wall and defense mechanisms. The high abundance of membrane proteins may indicate that the stress conditions increased the production of toxic metabolites, which can be eliminated from cells via pump systems [56]. The protein TolC is important for membrane structure and function in *E. coli*, as well as in the export of toxic molecules in gram-negative bacteria, and TolC mutants are impaired in detoxification and repair [57]. The proteins MdtA, MdtN, MdtB, and MdtC belong to the resistance-nodulation-division (RND) family of transporters, which play a role in drug resistance in Gram-negative bacteria and promote the efflux of a wide range of toxic compounds, including antibiotics and detergents [58,59]. The outer membrane protein OprM is part of a membrane protein complex capable of actively ejecting an assortment of harmful compounds and is most notably involved in multidrug-resistance in Gram-negative bacteria such as *Pseudomonas aeruginosa* [60]. Interestingly, genes encoding cation/multidrug efflux pumps are upregulated in cells of *Acetobacter aceti* grown in glucose, although the reasons are unknown [61]. In addition, it was already observed that strains of *Acidobacteria* have a large proportion of their genomes dedicated to transport [1]. Most of the genes are related to drug/metabolite transport; however, a broad range of substrate categories can be observed, suggesting that *Acidobacteria* may have advantages in nutrient uptake, especially in oligotrophic, nutrient-limited conditions [1].

KEGG metabolic pathway analysis of the upregulated proteins and transcripts demonstrated that no specific metabolic pathway was enhanced by the high sugar treatment. Nonetheless, a predominance of categories related to membrane protein complexes was observed, such as two-component systems, ABC transporters, and bacterial secretion systems. Closer examination of the proteins involved in carbon metabolism revealed the upregulation of proteins related to trehalose biosynthesis. Trehalose is an osmoprotectant disaccharide that accumulates in microorganisms under conditions of osmotic stress, and its role in cellular protection has been demonstrated in bacteria and yeast [62]. The expression of these enzymes indicates that the bacterial cells were reacting to the high sugar conditions by storing trehalose to increase their resistance to osmotic stress. The increased sugar concentration also has an effect on the expression of many proteins that are of unknown function. Lack of functional prediction is a general problem, with one-third of all genes in a genome having no assigned function at present [63]. In-depth analysis of those proteins might give further insights into the complex regulatory mechanisms, metabolic changes, or stress responses triggered by high sugar concentrations.

The upregulation of protein GWH15_11910, which is similar to a cellobiose phosphorylase, may confirm the use of cellobiose as a carbon source, since this enzyme catalyzes the phosphorolysis of the β-1,4-glucosidic bond of the disaccharide to produce α-D-glucose-1-phosphate and D-glucose [64]; however, no other carbon metabolism pathways were enhanced. Furthermore, the catabolic control protein CcpA was upregulated. The impact of this protein on cellular metabolism will be discussed later, as it promotes the repression of several metabolic pathways. The analysis of the downregulated transcripts indicated a decline in cell activity marked by reduced protein synthesis, carbohydrate metabolism, and cell division, consistent with the osmotic stress conditions imposed by the higher amount of sugar in the culture medium. The downregulated proteins included the enzyme anthranilate isomerase, which is involved in tryptophan biosynthesis [65]; RNase P, which is responsible for the production of the mature 5′ ends of precursor tRNAs and is essential for translation [66]; and the small subunit of the ribosome, 16S rRNA, which is fundamental for protein synthesis in prokaryotes [67]. As part of central carbon metabolism, the enzyme triosephosphate isomerase is vital for energy production, since it plays a key role in glycolysis, gluconeogenesis, and the pentose phosphate and Entner–Doudoroff pathways [68]. The tyrosine recombinase XerC has a significant role in chromosome segregation during cell division and is important for the maintenance of replicon stability in *E. coli* [69], and the chaperone hspA is a heat shock protein that is involved in the correct folding of proteins [70]. The thioredoxin TrxA is a small redox protein that also possesses a chaperone function [71]. The analysis of the downregulated proteins further revealed a slowing of metabolism, as the majority of the downregulated proteins were related to energy metabolism and cofactor biosynthesis, consistent with the downregulation of transcripts related to carbohydrate metabolism and cell division. Similarly, when exposed to osmotic stress, the metabolism of *E. coli* growing in minimal medium slows, and respiration and macromolecule synthesis are inhibited [72]. Osmotic pressure inhibits membrane functions, which could explain the expression of membrane stress signals observed in the present study. In addition, osmotic stress in *Sinorhizobium meliloti* promotes the repression of several functions of central metabolism and energy production systems [73]. Oxygen starvation might be responsible for part of the stress response produced by strain WH15 in the high sugar treatment [74]; however we did not observe upregulation of transcripts or proteins involved in the regulation of oxygen starvation response among the annotated proteins, and both treatments were exposed to the same oxygen conditions. An important protein impacting cell metabolism is the catabolic control protein CcpA, which was upregulated under high sugar conditions. This protein is involved in carbon catabolite repression (CCR), a regulatory process that—in the presence of a preferable carbon source—inhibits alternative metabolic pathways [75]. Although mainly found in Gram-positive bacteria, it has been suggested that Gram-negative bacteria might have CcpA-dependent CCR [76]. CCR regulates not only genes and operons involved in carbon metabolism, but also those involved in the metabolism of amino acids and nucleotides and in the synthesis of extracellular enzymes and secondary metabolites [77]. Consistent with this process, we observed the downregulation of several enzymes related to secondary carbon source hydrolysis, such as beta galactosidases, xylosidases, and galacturonases. Furthermore, enzymes related to amino acids and fatty acid metabolism and their corresponding KEGG pathways were repressed. One of the main repressed carbon pathways was the pentose phosphate (PP) pathway. The PP pathway is fundamental for the generation of NADPH molecules and biosynthetic intermediates that are essential for the production of fatty acids, glutamate, purines, histidine, and aromatic amino acids [78]. Therefore, downregulation of the PP pathway could also contribute to the general repression of cell metabolism.

Although many transcripts and proteins could not be identified due to missing information in the databases, the analysis of the present dataset demonstrates that the addition of a high sugar concentration in the culture medium of *Granulicella* sp. WH15 initially triggered a stress response. As part of this response, excretory functions were intensified to promote detoxification and inhibit the production of toxic compounds by bacterial metabolism; energy metabolism was repressed to reallocate resources towards maintenance instead of growth; and the production of the osmoprotectant trehalose was enhanced. Features such as tolerance to higher concentrations of carbon sources, the presence of several types of glycoside hydrolases, and a wide range of transporters, as observed in our study, might be fundamental characteristics for the development and prevalence of strains of *Acidobacteria* in soil environments. Data from disturbance experiments are important for understanding the behavior of microorganisms in response to environmental stresses, particularly for *Acidobacteria*, which is highly abundant in soil but whose soil ecology remains to be established. Further studies will improve our understanding of the mechanisms underlying the adjustment of the slow growth of bacterial strains under stress to higher growth rates.

## Figures and Tables

**Table 1:** Genomic statistics.

Genome	*Granulicella* spp. WH15
Size (bp)	4,673,153
G+C content (%)	60.7
Number of coding sequences	3939
Number of features in subsystems	1456
Number of RNA genes	51
Number of contigs	1

**Table 2:** Quantitative comparison of coding sequences, RNA and subsystems among species of *Granulicella.*

Genome	Size (bp)	G+C Content	Number of Coding Sequences	Number of Features	Number of Subsystems	Number of RNAs	ANI *
*Granulicella* spp. WH15	4,673,153	60.7	3871	1496	374	51	100
*Granulicella pectinivorans* [9]	4,439,413	61.2	3681	1066	302	41	73.32
*Granulicella mallensis* MP5ACTX8 [40]	6,237,577	57.9	5008	1662	394	50	73.03
*Granulicella tundricola* MP5ACTX9 [41]	5,503,984	60	4730	1526	361	49	74.12
*Granulicella rosea* DSM 18704 [9]	5,293,785	62.9	4515	864	250	55	75.32

*Average nucleotide identity percentage values between *Granulicella* strain WH15 and other available *Granulicella* strains’ whole genomes.

**Table 3:** Carbohydrate-Active Enzyme (CAZyme) categories observed in the WH15 genome.

CAZyme Family	Counts
Auxiliary activity (AA)	13
Carbohydrate binding module (CBM)	22
Carbohydrate esterase (CE)	41
Cohesin	1
Glycoside hydrolase (GH)	86
Glycosyl transferase (GT)	52
Polysaccharide lyase (PL)	2
**Total**	**217**

**Table 4:** Biosynthetic gene clusters reveled by analysis with ANTISMASH.

Cluster	Type	Most Similar Known Cluster
Cluster 2	putative	O-antigen BGC (19% of genes show similarity)
Cluster 3	Cf saccharide	
Cluster 4	t3pks-cf fatty acid	
Cluster 7	Cf saccharide	
Cluster 11	lassopeptide	
Cluster 13	bacteriocin	
Cluster 16	Cf saccharide	
Cluster 17	Cf fatty acid-terpene	Malleobactin BGC (11% of genes show similarity)
Cluster 19	t3pks	
Cluster 22	terpene	
Cluster 26	bacteriocin	
Cluster 27	Cf saccharide	
Cluster 28	putative	Thuggacin BGC (15% of genes show similarity)

Cf: possible cluster found with ClusterFinder algorithm from ANTISMASH; BCG: biosynthetic gene cluster; t3pks: type III polyketide synthase.

**Table 5:** Downregulated genes assigned to KEGG metabolic pathways.

KEGG Orthology	Gene	Related KEGG Pathways
K01817	*trpF*	Biosynthesis of antibiotics, secondary metabolites, and amino acids; metabolic pathways; phenylalanine, tyrosine, and tryptophan biosynthesis.
K01803	*tpiA*	Biosynthesis of antibiotics, secondary metabolites, and amino acids; metabolic pathways; phenylalanine, tyrosine, and tryptophan biosynthesis; microbial metabolism in diverse environments; carbon metabolism; inositol phosphate metabolism; fructose and mannose metabolism; glycolysis/gluconeogenesis.
K04043	*dnaK*	RNA degradation.
K01609	*trpC*	Biosynthesis of antibiotics, secondary metabolites, and amino acids; metabolic pathways; phenylalanine, tyrosine, and tryptophan biosynthesis.
K00033	*gndA*	Biosynthesis of antibiotics and secondary metabolites; metabolic pathways; microbial metabolism in diverse environments; carbon metabolism; phenylalanine, tyrosine, and tryptophan biosynthesis; glutathione metabolism; pentose phosphate pathway.

## Supplementary Materials

The following are available online at https://www.mdpi.com/2076-2607/8/2/244/s1, Figure S1: Cluster analysis of the proteome profile based on qualitative data in low and high sugar conditions., Figure S2: Expression pattern of proteins under high and low sugar cultivation of *Granulicella* sp. WH15, Figure S3: TigrFam roles of the differentially expressed proteins, excluding proteins with unknown function, Figure S4: General overview of up (red) and downregulated (blue) metabolic pathways based on KEGG analysis of proteome, Table S1: growth of strain *Granulicella* sp. WH15 in culture media supplemented with different carbon sources, Table S2: Total number of transcripts reads per sample in low and high sugar conditions, Table S3: Differentially up and down regulated transcripts in high sugar treatment, Table S4: Differentially upregulated proteins in high sugar treatment, Table S5: Number of proteins assigned to KEGG metabolic pathways, Table S6: Differentially downregulated proteins in high sugar treatment.
