# Supplementary material for: Responses of Acidobacteria Granulicella sp. WH15 to High Carbon Revealed by Integrated Omics Analyses
Source: Microorganisms. 2020 Feb 12;8(2):244. doi: 10.3390/microorganisms8020244 (PMC7074687; doi:10.3390/microorganisms8020244)
Supplement: Supplementary file 1 [file microorganisms-08-00244-s001.pdf]

Supplementary material

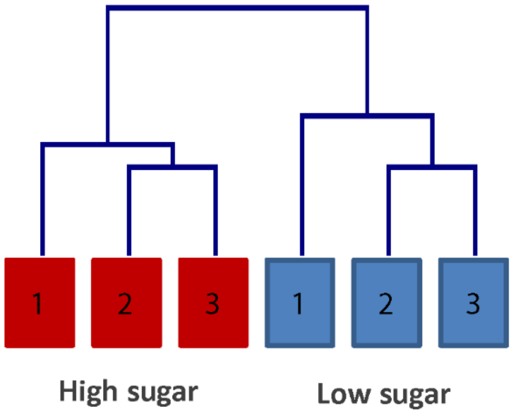

Figure S1. Cluster analysis of the proteome profile based on qualitative data in low and high sugar conditions.

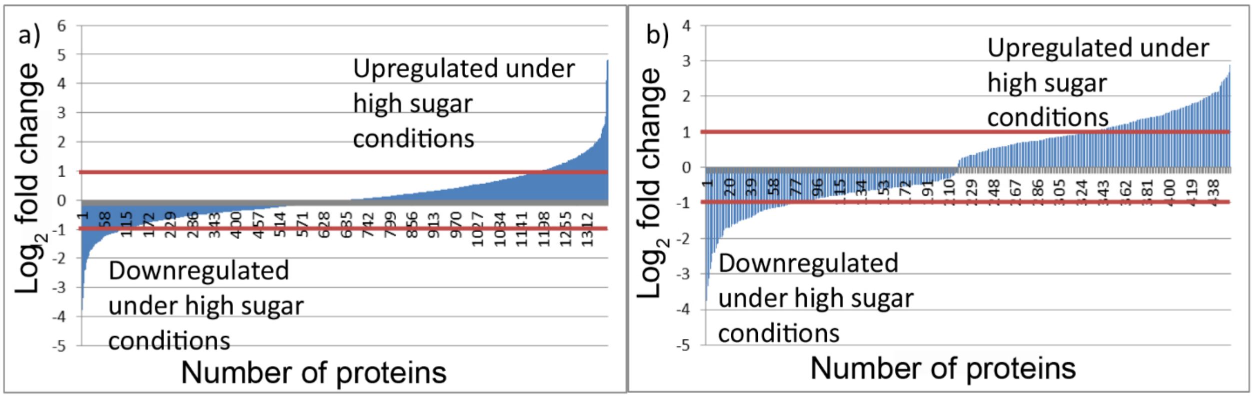

Figure S2. Expression pattern of proteins under high and low sugar cultivation of *Granulicella* sp. WH15 a) All proteins identified in at least two out of three replicates (excluding on/off proteins). b) Only proteins with significant change t-test p=0.01. 2fold change is indicated by a red line.

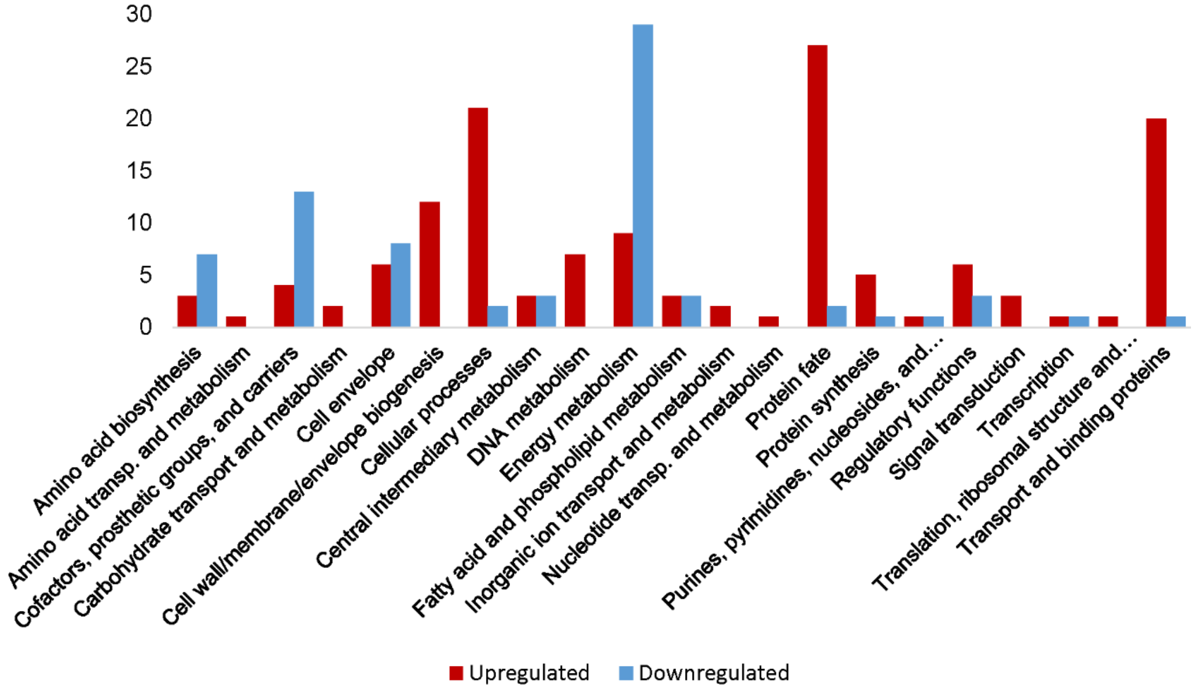

Figure S3. TigrFam roles of the differentially expressed proteins, excluding proteins with unknown function.

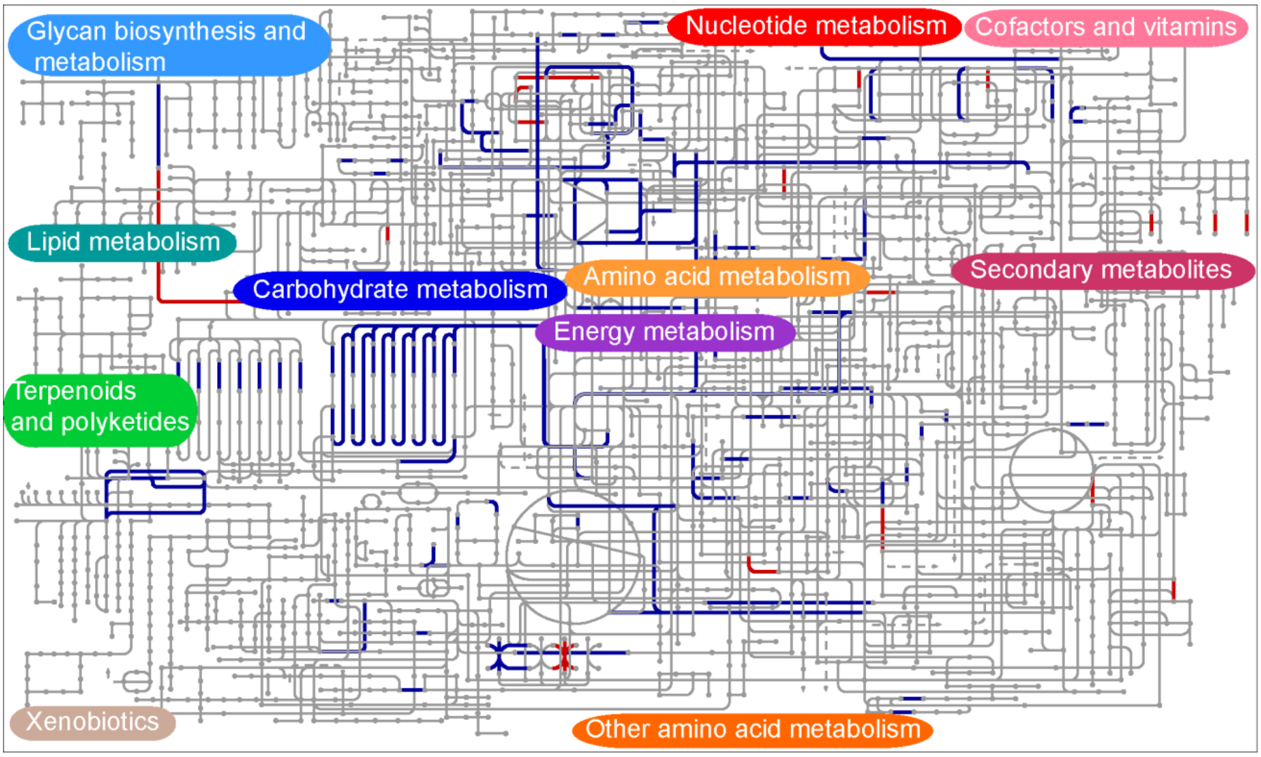

Figure S4. General overview of up (red) and downregulated (blue) metabolic pathways based on KEGG analysis of proteome.

Table S1. growth of strain *Granulicella* sp. WH15 in culture media supplemented with different carbon sources.

Carbon Source	Growth
Pectin	-
Glycogen	-
Glucosamine	-
Cellulose	-
D-glucose	+
D-galactose	+
D-mannose	+
D-xylose	+
L-arabinose	+
L-rhamnose	+
D-galacturonic acid	-
Cellobiose	+
D-lactose	+
Sucrose	+

+=positive growth; -=No growth.

Table S2. Total number of transcripts reads per sample in low and high sugar conditions**.**

Sample ID	Total Number of Reads
Low sugar (1)	15,731,147
Low sugar (2)	12,624,878
Low sugar (3)	11,080,985
High sugar (1)	11,138,128
High sugar (2)	9,322,795
High sugar (3)	10,071,593

Table S3. Differentially up and down regulated transcripts in high sugar treatment.

ORF	Annotation	Log_2FC
GWH15_14040	hypothetical protein	3.71
GWH15_06005	hypothetical protein	3.12
GWH15_00285	tRNA-Asn(gtt)	2.74
GWH15_06010	hypothetical protein	2.70
GWH15_14055	hypothetical protein	2.66
GWH15_14060	hypothetical protein	2.18
GWH15_14100	hypothetical protein	2.00
GWH15_08445	hypothetical protein	1.94
GWH15_05955	hypothetical protein	1.93
GWH15_14070	gfo4	1.92
GWH15_16895	tRNA-Asp(gtc)	1.72
GWH15_17685	HigA	1.71
GWH15_18230	hypothetical protein	1.67
GWH15_01965	LytR	1.67
GWH15_18255	hypothetical protein	1.60
GWH15_06405	hypothetical protein	1.59
GWH15_02080	hypothetical protein	1.54
GWH15_10070	hypothetical protein	1.47
GWH15_07155	hypothetical protein	1.41
GWH15_03690	hypothetical protein	1.37
GWH15_14050	SigW	1.35
GWH15_05960	hypothetical protein	1.32
GWH15_05985	hypothetical protein	1.26
GWH15_17690	HigB-1	1.17
GWH15_11830	hypothetical protein	1.13
GWH15_14160	hypothetical protein	1.10
GWH15_06410	hypothetical protein	1.06
GWH15_04600	putative peroxiredoxin	1.04
GWH15_17625	hypothetical protein	-1.03
GWH15_00710	hypothetical protein	-1.06
GWH15_00505	RpoD	-1.09
GWH15_16220	hypothetical protein	-1.19
GWH15_14410	Lon protease 2	-1.20
GWH15_08600	hypothetical protein	-1.21
GWH15_14215	hypothetical protein	-1.22
GWH15_10510	hypothetical protein	-1.29
GWH15_00210	RNaseP_bact_a	-1.30
GWH15_01535	gndA	-1.33
GWH15_00720	hypothetical protein	-1.33
GWH15_01770	23S ribosomal RNA	-1.34
GWH15_19395	hypothetical protein	-1.34
GWH15_00785	trpC	-1.39
GWH15_04765	XerC	-1.44
GWH15_01625	DnaK	-1.45
GWH15_11560	hypothetical protein	-1.47
GWH15_07810	SsrA	-1.53
GWH15_15305	glpF	-1.63
GWH15_08540	hypothetical protein	-1.75
GWH15_12250	trxA	-1.80
GWH15_01755	16S ribosomal RNA	-1.94

GWH15_12725	hypothetical protein	-2.06
GWH15_08685	XerC	-2.09
GWH15_16995	tpiA	-2.10
GWH15_00715	hypothetical protein	-2.26
GWH15_06890	XerC	-2.50
GWH15_00885	hspA	-2.60
GWH15_00780	trpF	-2.80
GWH15_19400	hypothetical protein	-3.05

Table S4. Differentially upregulated proteins in high sugar treatment.

ORF	Description	$\log_2$ FC
GWH15_02420	Hypothetical protein	4
GWH15_16935	7-carboxy-7-deazaguanine synthase	4
GWH15_15250	ABC transporter ATP-binding protein ytrb	4
GWH15_08630	AI-2 transport protein tqsa	4
GWH15_07165	Aminopeptidase ypdf	4
GWH15_00460	Amylopullulanase	4
GWH15_08480	Anti-sigma-K factor rska	4
GWH15_08415	Apolipoprotein N-acyltransferase	4
GWH15_00205	Band 7 protein	4
GWH15_05535	Beta-barrel assembly-enhancing protease	4
GWH15_17125	Carbohydrate acetyl esterase/feruloyl esterase	4
GWH15_11910	Carbohydrate transport and metabolism-Glycosyltransferase 36	4
GWH15_15690	Carboxy-terminal processing protease ctpa	4
GWH15_09810	Catabolite control protein A	4
GWH15_18425	CDP-diacylglycerol--glycerol-3-phosphate 3-phosphatidyltransferase	4
GWH15_19395	Cell wall/membrane/envelope biogenesis asma family	4
GWH15_01995	Cell wall/membrane/envelope biogenesis-asma family	4
GWH15_09540	Cna B-type protein-transport	4
GWH15_18835	Cobalt-zinc-cadmium resistance protein czca	4
GWH15_18685	Conserved protein-pfam:duf403	4
GWH15_18690	Conserved protein-pfam:duf403	4
GWH15_09565	Cyclic pyranopterin monophosphate synthase 1	4
GWH15_10075	Cytochrome c oxidase subunit 2	4
GWH15_09055	Cytochrome c-type biogenesis protein ccmf	4
GWH15_10475	Diaminopimelate decarboxylase	4
GWH15_00815	Diguanylate cyclase dosc	4
GWH15_00520	DNA primase	4
GWH15_13725	DNA-directed RNA polymerase subunit omega	4
GWH15_03780	D-ribose-binding periplasmic protein	4
GWH15_02260	Endonuclease muts2	4
GWH15_00620	Hypothetical protein	4
GWH15_01680	Exopolyphosphatase	4
GWH15_18735	FAD-dependent decaprenylphosphoryl-beta-D-ribofuranose 2-oxidase	4
GWH15_08515	Ferrous iron permease efeu	4
GWH15_00500	Glycogen synthase	4
GWH15_06205	Guanine deaminase	4
GWH15_12695	H(+)/Cl(-) exchange transporter clca	4
GWH15_00225	Hopanoid biosynthesis associated glycosyl transferase protein hpni	4
GWH15_19130	HTH-type transcriptional repressor fabr	4
GWH15_12380	Hypothetical protein	4
GWH15_00410	Hypothetical protein	4

GWH15_00175	Hypothetical protein	4
GWH15_01300	Hypothetical protein	4
GWH15_03025	Hypothetical protein	4
GWH15_04195	Hypothetical protein	4
GWH15_04880	Hypothetical protein	4
GWH15_05505	Hypothetical protein	4
GWH15_05985	Hypothetical protein	4
GWH15_06025	Hypothetical protein	4
GWH15_06760	Hypothetical protein	4
GWH15_07215	Hypothetical protein	4
GWH15_08350	Hypothetical protein	4
GWH15_13120	Hypothetical protein	4
GWH15_15045	Hypothetical protein	4
GWH15_15255	Hypothetical protein	4
GWH15_16300	Hypothetical protein	4
GWH15_18130	Hypothetical protein	4
GWH15_02890	Hypothetical protein	4
GWH15_07610	Inorganic ion transport and metabolism-tonb-dependent Receptor	4
GWH15_08030	Inorganic ion transport and metabolism-tonb-dependent Receptor	4
GWH15_15415	Inorganic ion transport and metabolism-tonb-dependent Receptor	4
GWH15_00615	Tonb-dependent heme/hemoglobin receptor family protein	4
GWH15_15365	Isoaspartyl dipeptidase	4
GWH15_03790	L-asparaginase 2	4
GWH15_11775	Lexa repressor	4
GWH15_12485	Lipid transport and metabolism-desaturase	4
GWH15_15780	LPS-assembly lipoprotein lpte	4
GWH15_13770	LPS-assembly protein lptd	4
GWH15_15340	Macrolide export ATP-binding/permease protein macb	4
GWH15_15475	Macrolide export ATP-binding/permease protein macb	4
GWH15_03815	Macrolide export protein maca	4
GWH15_12245	Magnesium and cobalt efflux protein corc	4
GWH15_06965	Mannosylfructose-phosphate synthase	4
GWH15_16655	Metallo-beta-lactamase family	4
GWH15_11300	Multidrug resistance protein mdtb	4
GWH15_12080	Multidrug resistance protein mdtb	4
GWH15_19560	Multidrug resistance protein mdtc	4
GWH15_12085	Multidrug resistance protein mdtc	4
GWH15_08245	NADH-quinone oxidoreductase subunit M	4
GWH15_15355	N-formyl-4-amino-5-aminomethyl-2-methylpyrimidine deformylase	4
GWH15_19200	N-formyl-4-amino-5-aminomethyl-2-methylpyrimidine deformylase	4
GWH15_08145	Nuclear protein SET	4
GWH15_15850	Nucleoside permease nupx	4
GWH15_13750	Nucleotide-binding protein	4
GWH15_14450	Outer membrane protein assembly factor bama	4
GWH15_03820	Outer membrane protein oprm	4
GWH15_11310	Outer membrane protein oprm	4
GWH15_12090	Outer membrane protein oprm	4
GWH15_16030	Outer membrane protein oprm	4
GWH15_18825	Outer membrane protein oprm	4
GWH15_17110	Penicillin-binding protein 2	4
GWH15_16260	Pfam:DUF811	4
GWH15_14525	PGL/p-HBAD biosynthesis glycosyltransferase	4

GWH15_05440	Phytochrome-like protein cph1	4
GWH15_05490	Poly-beta-1,6-N-acetyl-D-glucosamine synthase	4
GWH15_06970	Polysaccharide export protein	4
GWH15_14210	Polysialic acid transport protein kpsd	4
GWH15_09965	Protein ycei	4
GWH15_15350	Putative ABC transporter ATP-binding protein ykny	4
GWH15_01450	Putative ctpa-like serine protease	4
GWH15_13740	Putative multidrug export ATP-binding/permease protein	4
GWH15_16305	Putative NTE family protein	4
GWH15_17565	Putative thiazole biosynthetic enzyme	4
GWH15_15010	Putative tonb-dependent receptor bfrd	4
GWH15_14825	Putative zinc metalloprotease	4
GWH15_13800	Putative zinc metalloprotease Rip3	4
GWH15_13795	Ribosomal RNA small subunit methyltransferase D	4
GWH15_14980	Ribosomal RNA small subunit methyltransferase H	4
GWH15_16290	Rlpa-like protein	4
GWH15_09495	Sensor protein zras	4
GWH15_01965	Sensory transduction protein lytr	4
GWH15_19165	Signal transduction mechanisms Serine Threonine protein kinase	4
GWH15_09570	Squalene-hopene cyclase	4
GWH15_12830	Thiol-disulfide oxidoreductase resa	4
GWH15_05860	Tonb-dependent Receptor	4
GWH15_05950	Tonb-dependent Receptor	4
GWH15_12055	Tonb-dependent Receptor	4
GWH15_14280	Tonb-dependent Receptor	4
GWH15_03535	Tonb-dependent receptor plug	4
GWH15_19020	Tonb-dependent receptor plug	4
GWH15_09325	Transcriptional activator cadc	4
GWH15_16620	Transcriptional regulatory protein ypdb	4
GWH15_14440	Translocation and assembly module tamb	4
GWH15_13920	Trehalase	4
GWH15_01390	Two component, sigma54 specific, transcriptional regulator, Fis family	4
GWH15_01570	Type II secretion system protein F	4
GWH15_06185	UDP-glucose:undecaprenyl-phosphate glucose-1-phosphate transferase	4
GWH15_11890	Undecaprenyl-phosphate 4-deoxy-4-formamido-L-arabinose transferase	4
GWH15_19395	Hypothetical protein	4
GWH15_03080	Putative mycofactocin radical SAM maturase mftc	2.88
GWH15_17755	Tonb-dependent receptor plug	2.68
GWH15_10545	Type II secretion system protein D	2.60
GWH15_17590	Hypothetical protein	2.54
GWH15_10175	Catalase-related peroxidase	2.52
GWH15_07490	Multidrug resistance protein mdtn	2.48
GWH15_07480	Outer membrane efflux protein bepc	2.44
GWH15_05765	Tonb-dependent receptor plug	2.42
GWH15_11620	Hypothetical protein	2.32
GWH15_14285	Tonb-dependent Receptor	2.28
GWH15_11915	Lipid A export ATP-binding/permease protein msba	2.13
GWH15_15410	Tonb-dependent Receptor	2.12
GWH15_13055	Putative phospholipid ABC transporter-binding protein mlad	2.12
GWH15_16040	Multidrug resistance protein mdtc	2.11
GWH15_00900	N-formyl-4-amino-5-aminomethyl-2-methylpyrimidine deformylase	2.10
GWH15_01960	Hypothetical protein	2.08

GWH15_13535	Hypothetical protein	2.07
GWH15_01785	TM2 domain	2.02
GWH15_13930	Trehalose synthase/amylase tres	2.02
GWH15_16185	Hypothetical protein	1.99
GWH15_04655	ABC transporter, permease	1.97
GWH15_17630	BON domain	1.96
GWH15_12075	Multidrug resistance protein mdta	1.95
GWH15_18985	Trehalose-phosphate synthase	1.95
GWH15_05290	Hypothetical protein	1.90
GWH15_15725	ATP-dependent zinc metalloprotease ftsh	1.89
GWH15_13030	Mechanosensitive ion channel	1.89
GWH15_08035	Putative membrane protein mmpl3	1.85
GWH15_00705	Outer membrane protein assembly factor bama	1.83
GWH15_19570	Outer membrane protein tolc	1.83
GWH15_01695	Aminopeptidase N	1.83
GWH15_19225	Sporulation kinase E	1.82
GWH15_13285	Thymidylate kinase	1.81
GWH15_11185	Uvrabc system protein A	1.81
GWH15_01225	Toluene efflux pump outer membrane protein ttgf	1.76
GWH15_15395	Peptidase M14, carboxypeptidase A	1.75
GWH15_02935	Multidrug resistance protein mdtb	1.73
GWH15_02030	NADH dehydrogenase-like protein yjld	1.73
GWH15_02940	Multidrug resistance protein mdta	1.72
GWH15_15995	DNA mismatch repair protein mutl	1.71
GWH15_03075	ABC transporter permease ytrf	1.69
GWH15_15795	Disulphide bond corrector protein dsbc	1.68
GWH15_19215	Tonb-dependent receptor plug	1.67
GWH15_05980	Tonb-dependent Receptor	1.66
GWH15_06975	Tyrosine-protein kinase ywqd	1.64
GWH15_04000	Tonb-dependent receptor plug	1.63
GWH15_15525	ABC transporter ATP-binding protein nata	1.62
GWH15_14150	Hypothetical protein	1.61
GWH15_01740	Succinyl-diaminopimelate desuccinylase	1.60
GWH15_06990	Hypothetical protein	1.60
GWH15_14235	Tonb-dependent Receptor	1.60
GWH15_04050	Hypothetical protein	1.59
GWH15_02945	Decaprenyl-phosphate phosphoribosyltransferase	1.57
GWH15_07680	Catalase-peroxidase	1.54
GWH15_01780	Hypothetical protein	1.54
GWH15_14045	Putative oxidoreductase catd	1.53
GWH15_12475	Pca regulon regulatory protein	1.51
GWH15_02075	Hypothetical protein	1.48
GWH15_01335	Hypothetical protein	1.48
GWH15_12385	Ribosomal protein S12 methylthiotransferase rimo	1.47
GWH15_00530	Outer membrane protein domain-containing protein	1.47
GWH15_07050	Multidrug export protein emra	1.45
GWH15_19055	Imidazolonepropionase	1.44
GWH15_12370	Putative efflux system component yknx	1.44
GWH15_09425	Efflux pump membrane transporter bepe	1.44
GWH15_18435	Anthranilate synthase component 1	1.43
GWH15_14490	DNA polymerase/3'-5' exonuclease polx	1.42
GWH15_05965	Tonb-dependent Receptor	1.42

GWH15_12430	Putative phospholipid ABC transporter permease protein mlae	1.41
GWH15_16035	Multidrug resistance protein mdta	1.41
GWH15_17465	Outer membrane protein assembly factor bamd	1.40
GWH15_02400	Tonb family	1.40
GWH15_02910	Hypothetical protein	1.39
GWH15_09000	Putative membrane protein	1.37
GWH15_15800	Thiol-disulfide oxidoreductase resa	1.37
GWH15_12290	Outer membrane lipoprotein Omp16	1.36
GWH15_09420	Outer membrane protein oprm	1.36
GWH15_17035	Glutathione synthase ribosomal protein s6 modification	1.36
GWH15_05770	Prolyl tripeptidyl peptidase	1.35
GWH15_13925	Alpha-1,4-glucan:maltose-1-phosphate maltosyltransferase 1	1.34
GWH15_02605	Methionine aminopeptidase 1	1.33
GWH15_08560	4,4'-diaponeurosporenoate glycosyltransferase	1.30
GWH15_11595	Putative mscs family protein ykut	1.30
GWH15_07225	Hypothetical protein	1.29
GWH15_13275	Hypothetical protein	1.28
GWH15_07045	Outer membrane protein oprm	1.28
GWH15_11845	Vitamin B12 transporter btub	1.27
GWH15_19245	Biopolymer transport protein exbd	1.26
GWH15_01440	Glycosyl transferase family	1.23
GWH15_12815	Tonb-dependent receptor plug	1.23
GWH15_06900	BON domain	1.22
GWH15_03995	Feruloyl esterase	1.22
GWH15_12700	Tonb family	1.22
GWH15_18875	Tonb-dependent receptor plug	1.21
GWH15_13410	HTH-type transcriptional regulator lutr	1.19
GWH15_09020	Hypothetical protein	1.19
GWH15_04110	2-dehydro-3-deoxy-D-gluconate 5-dehydrogenase	1.18
GWH15_02670	Translation initiation factor IF-2	1.18
GWH15_18510	Biodegradative arginine decarboxylase	1.17
GWH15_05295	Dispase autolysis-inducing protein	1.15
GWH15_19675	Membrane protein insertase yidc	1.15
GWH15_07605	Response regulator protein vrar	1.15
GWH15_00730	30S ribosomal protein S1	1.14
GWH15_02465	30S ribosomal protein S12	1.13
GWH15_12305	Hypothetical protein	1.13
GWH15_19615	DNA-binding protein HU	1.12
GWH15_17135	Rod shape-determining protein mreb	1.11
GWH15_03785	L-asparaginase 2	1.11
GWH15_15505	Energy-dependent translational throttle protein etta	1.09
GWH15_18520	Exodeoxyribonuclease 7 large subunit	1.08
GWH15_16085	Hypothetical protein	1.07
GWH15_14870	Trna threonylcarbamoyladenosine biosynthesis protein tsae	1.07
GWH15_03680	Vitamin B12-dependent ribonucleoside-diphosphate reductase	1.06
GWH15_05990	Hypothetical protein	1.06
GWH15_12285	Protein tolb	1.05
GWH15_11850	Hypothetical protein	1.05
GWH15_01330	Putative nicotinate-nucleotide pyrophosphorylase [carboxylating]	1.04
GWH15_11780	Hypothetical protein	1.03
GWH15_19025	Quinate/shikimate dehydrogenase (quinone)	1.03
GWH15_04580	Methionine synthase	1.02

GWH15_07455	Protein translocase subunit seca	1.01

Table S5. Number of proteins assigned to KEGG metabolic pathways.

KEGG Metabolic Pathways	Upregulated	Downregulated
Metabolic pathways	15	56
Biosynthesis of secondary metabolites	6	22
Biosynthesis of antibiotics	3	19
Microbial metabolism in diverse environments	1	14
Carbon metabolism	1	12
Biosynthesis of amino acids	3	11
Fatty acid metabolism	1	8
Lipopolysaccharide biosynthesis	0	6
Fatty acid biosynthesis	0	5
Butanoate metabolism	0	5
Propanoate metabolism	0	5
Valine, leucine and isoleucine degradation	0	5
Starch and sucrose metabolism	2	4
Galactose metabolism	0	4
Amino sugar and nucleotide sugar metabolism	0	4
Glyoxylate and dicarboxylate metabolism	1	4
Fatty acid degradation	0	4
Phenylalanine, tyrosine and tryptophan biosynthesis	1	4
Pantothenate and CoA biosynthesis	0	4
Benzoate degradation	0	4
Pentose phosphate pathway	0	4
Other glycan degradation	0	3
Quorum sensing	3	3
Novobiocin biosynthesis	0	3
Phenylalanine metabolism	1	3
Cysteine and methionine metabolism	1	3
Methane metabolism	0	3
2-Oxocarboxylic acid metabolism	0	3
Cyanoamino acid metabolism	0	2
Folate biosynthesis	0	2
Glycolysis / Gluconeogenesis	0	2
Tyrosine metabolism	0	2
Fructose and mannose metabolism	0	2
Histidine metabolism	0	2
Pentose and glucuronate interconversions	0	2
Pyruvate metabolism	0	2
Nitrogen metabolism	0	2
Arginine biosynthesis	0	2
Synthesis and degradation of ketone bodies	0	2
Valine, leucine and isoleucine biosynthesis	0	2
beta-Alanine metabolism	0	2
Riboflavin metabolism	0	2
Geraniol degradation	0	2
Alanine, aspartate and glutamate metabolism	0	2
Biotin metabolism	0	1
ABC transporters	4	1
Sulfur metabolism	0	1
Bacterial chemotaxis	2	1

Arginine and proline metabolism	1	1
Thiamine metabolism	0	1
Glycine, serine and threonine metabolism	0	1
Xylene degradation	0	1
Purine metabolism	2	1
Glutathione metabolism	2	1
Oxidative phosphorylation	3	1
Cationic antimicrobial peptide (CAMP) resistance	1	1
Degradation of aromatic compounds	0	1
Two-component system	9	1
C5-Branched dibasic acid metabolism	0	1
Pyrimidine metabolism	1	1
RNA degradation	0	1
Terpenoid backbone biosynthesis	0	1
alpha-Linolenic acid metabolism	0	1
Sphingolipid metabolism	1	1
Selenocompound metabolism	1	1
Glycerophospholipid metabolism	1	1
D-Glutamine and D-glutamate metabolism	0	1
Peptidoglycan biosynthesis	0	1
Chlorocyclohexane and chlorobenzene degradation	0	1
Inositol phosphate metabolism	0	1
Biosynthesis of unsaturated fatty acids	1	0
Lysine biosynthesis	1	0
Sesquiterpenoid and triterpenoid biosynthesis	1	0
beta-Lactam resistance	2	0
Bacterial secretion system	4	0
Tryptophan metabolism	2	0
Ribosome	2	0
Protein export	2	0
Nucleotide excision and repair	1	0
Mismatch repair	2	0
One carbon pool by folate	1	0
DNA replication	1	0
RNA polymerase	1	0
Nicotinate and nicotinamide metabolism	1	0
Phenazine biosynthesis	1	0

Table S6. Differentially downregulated proteins in high sugar treatment.

ORF	Description	log_2 FC
GWH15_02345	3-isopropylmalate dehydratase small subunit 1	-4.00
GWH15_14090	Formate dehydrogenase	-4.00
GWH15_00995	Glutamate synthase [NADPH] small chain	-4.00
GWH15_00610	Glycerate dehydrogenase	-4.00
GWH15_17605	Homoserine O-acetyltransferase	-4.00
GWH15_06475	1-deoxy-D-xylulose-5-phosphate synthase	-4.00
GWH15_06375	4-hydroxythreonine-4-phosphate dehydrogenase 2	-4.00
GWH15_01205	Dephospho-coa kinase	-4.00
GWH15_08325	L-2,4-diaminobutyrate decarboxylase	-4.00
GWH15_10235	Multifunctional cyclase-dehydratase-3-O-methyl transferase tcmn	-4.00
GWH15_11220	Phosphomethylpyrimidine synthase	-4.00
GWH15_18315	Phosphopantetheine adenylyltransferase	-4.00

GWH15_07695	Pyrroloquinoline-quinone synthase	-4.00
GWH15_02155	Riboflavin biosynthesis protein ribd	-4.00
GWH15_18275	3-deoxy-manno-octulosonate cytidylyltransferase	-4.00
GWH15_01640	Acyl-[acyl-carrier-protein]--UDP-N-acetylglucosamine O-acyltransferase	-4.00
GWH15_15135	Lipopolysaccharide heptosyltransferase 1	-4.00
GWH15_15130	Tetraacyldisaccharide 4'-kinase	-4.00
GWH15_08130	UDP-3-O-acylglucosamine N-acyltransferase	-4.00
GWH15_12280	UDP-N-acetylenolpyruvoylglucosamine reductase	-4.00
GWH15_06130	Acetyl-coa:oxalate coa-transferase	-4.00
GWH15_07765	Phenylacetaldehyde dehydrogenase	-4.00
GWH15_11565	Biphenyl dioxygenase system ferredoxin subunit	-4.00
GWH15_11410	N-acetylglucosamine-6-phosphate deacetylase	-4.00
GWH15_07550	1,5-anhydro-D-fructose reductase	-4.00
GWH15_13480	2-dehydro-3-deoxy-D-gluconate 5-dehydrogenase	-4.00
GWH15_07735	2-hydroxy-3-oxopropionate reductase	-4.00
GWH15_17540	2-methylcitrate dehydratase	-4.00
GWH15_17545	2-methylcitrate synthase	-4.00
GWH15_05100	5'-nucleotidase	-4.00
GWH15_07745	Beta-galactosidase bgab	-4.00
GWH15_13400	Beta-xylosidase	-4.00
GWH15_07310	Inosose dehydratase	-4.00
GWH15_10460	NADH-quinone oxidoreductase subunit I	-4.00
GWH15_09940	Pyrethroid hydrolase	-4.00
GWH15_06155	Ribulose bisphosphate carboxylase-like protein 2	-4.00
GWH15_08005	Sorbitol dehydrogenase	-4.00
GWH15_06480	Transketolase 2	-4.00
GWH15_18410	D-xylose 1-dehydrogenase	-4.00
GWH15_03275	Release factor glutamine methyltransferase	-4.00
GWH15_08570	6-carboxy-5,6,7,8-tetrahydropterin synthase	-4.00
GWH15_06145	Hypothetical protein	-4.00
GWH15_16100	Hypothetical protein	-4.00
GWH15_01830	Transcriptional regulatory protein yycf	-4.00
GWH15_13365	2-keto-3-deoxy-L-fuconate dehydrogenase	-4.00
GWH15_11615	3',5'-cyclic adenosine monophosphate phosphodiesterase cpda	-4.00
GWH15_11945	4-hydroxy-4-methyl-2-oxoglutarate aldolase	-4.00
GWH15_17960	4-hydroxy-4-methyl-2-oxoglutarate aldolase	-4.00
GWH15_19220	6'''-hydroxyparomomycin C oxidase	-4.00
GWH15_05265	Acetylxylan esterase	-4.00
GWH15_15430	Acyl-coa dehydrogenase	-4.00
GWH15_16250	Acyl-coa dehydrogenase	-4.00
GWH15_03975	Acyl-coenzyme A thioesterase paai	-4.00
GWH15_17610	Beta-barrel assembly-enhancing protease	-4.00
GWH15_12730	Cellulose synthase operon protein C	-4.00
GWH15_06620	D-galactonate dehydratase	-4.00
GWH15_17965	D-galactonate dehydratase	-4.00
GWH15_11940	D-galactonate dehydratase family member	-4.00
GWH15_02130	Endonuclease 4	-4.00
GWH15_00665	Endo-polygalacturonase	-4.00
GWH15_17915	Exo-poly-alpha-D-galacturonosidase	-4.00
GWH15_09450	Glutamate-pyruvate aminotransferase alaa	-4.00
GWH15_02870	Hydroxymethylglutaryl-coa lyase yngg	-4.00
GWH15_08530	Hydroxypyruvate isomerase	-4.00

GWH15_00145	Hypothetical protein	-4.00
GWH15_00585	Hypothetical protein	-4.00
GWH15_02685	Hypothetical protein	-4.00
GWH15_03305	Hypothetical protein	-4.00
GWH15_03765	Hypothetical protein	-4.00
GWH15_03835	Hypothetical protein	-4.00
GWH15_03840	Hypothetical protein	-4.00
GWH15_05465	Hypothetical protein	-4.00
GWH15_06125	Hypothetical protein	-4.00
GWH15_06295	Hypothetical protein	-4.00
GWH15_06310	Hypothetical protein	-4.00
GWH15_06330	Hypothetical protein	-4.00
GWH15_06345	Hypothetical protein	-4.00
GWH15_06600	Hypothetical protein	-4.00
GWH15_07205	Hypothetical protein	-4.00
GWH15_07540	Hypothetical protein	-4.00
GWH15_07555	Hypothetical protein	-4.00
GWH15_07565	Hypothetical protein	-4.00
GWH15_07740	Hypothetical protein	-4.00
GWH15_07750	Hypothetical protein	-4.00
GWH15_07795	Hypothetical protein	-4.00
GWH15_07975	Hypothetical protein	-4.00
GWH15_08075	Hypothetical protein	-4.00
GWH15_08600	Hypothetical protein	-4.00
GWH15_08945	Hypothetical protein	-4.00
GWH15_09720	Hypothetical protein	-4.00
GWH15_10775	Hypothetical protein	-4.00
GWH15_12310	Hypothetical protein	-4.00
GWH15_12655	Hypothetical protein	-4.00
GWH15_12725	Hypothetical protein	-4.00
GWH15_13405	Hypothetical protein	-4.00
GWH15_13455	Hypothetical protein	-4.00
GWH15_13460	Hypothetical protein	-4.00
GWH15_15770	Hypothetical protein	-4.00
GWH15_16360	Hypothetical protein	-4.00
GWH15_16925	Hypothetical protein	-4.00
GWH15_17970	Hypothetical protein	-4.00
GWH15_18790	Hypothetical protein	-4.00
GWH15_19005	Hypothetical protein	-4.00
GWH15_11130	Hypothetical protein	-4.00
GWH15_09830	Hypothetical protein	-4.00
GWH15_06095	Hypothetical protein	-4.00
GWH15_17550	Methylisocitrate lyase	-4.00
GWH15_05095	Methylxanthine N1-demethylase ndma	-4.00
GWH15_00990	NAD-dependent dihydropyrimidine dehydrogenase subunit prea	-4.00
GWH15_13220	Phosphoribosyl-AMP cyclohydrolase	-4.00
GWH15_00845	Prephenate dehydrogenase	-4.00
GWH15_18405	Putative 3-hydroxybutyryl-coa dehydrogenase	-4.00
GWH15_02095	Putative formate dehydrogenase	-4.00
GWH15_12060	Putative N-succinyldiaminopimelate aminotransferase dapc	-4.00
GWH15_12955	Putative prophage major tail sheath protein	-4.00
GWH15_17805	Putative succinyl-coa:3-ketoacid coenzyme A transferase subunit B	-4.00

GWH15_03720	Reducing end xylose-releasing exo-oligoxylanase	-4.00
GWH15_09815	Retaining alpha-galactosidase	-4.00
GWH15_15040	Short-chain-enoyl-coa hydratase	-4.00
GWH15_13515	UDP-glucose 4-epimerase	-4.00
GWH15_07980	Hypothetical protein	-3.77
GWH15_07970	Hypothetical protein	-3.36
GWH15_07865	Putative acyl-coa dehydrogenase	-3.12
GWH15_07780	Gluconolactonase	-2.85
GWH15_07855	Putative 3-hydroxyacyl-coa dehydrogenase	-2.66
GWH15_13495	Virginiamycin B lyase	-2.42
GWH15_17530	Hypothetical protein	-2.40
GWH15_06300	Beta-galactosidase bgaa	-2.40
GWH15_07860	3-ketoacyl-coa thiolase	-2.28
GWH15_06320	Hypothetical protein	-2.16
GWH15_01715	Electron transfer flavoprotein subunit beta	-2.13
GWH15_18430	Glutamate dehydrogenase	-2.01
GWH15_07990	Extracellular exo-alpha-L-arabinofuranosidase	-1.97
GWH15_08000	Gluconate 5-dehydrogenase	-1.96
GWH15_06150	Non-reducing end beta-L-arabinofuranosidase	-1.91
GWH15_17820	Hypothetical protein	-1.77
GWH15_08445	Hypothetical protein	-1.73
GWH15_01710	Electron transfer flavoprotein subunit alpha	-1.71
GWH15_18330	Hypothetical protein	-1.71
GWH15_13235	Histidinol-phosphate aminotransferase 2	-1.70
GWH15_13420	Protein tolb	-1.70
GWH15_07965	Serine/threonine-protein kinase pknd	-1.69
GWH15_14310	Dihydroxy-acid dehydratase	-1.61
GWH15_07530	Hypothetical protein	-1.60
GWH15_03715	Hypothetical protein	-1.60
GWH15_11790	Metallo-beta-lactamase L1	-1.59
GWH15_09775	NAD-dependent malic enzyme	-1.58
GWH15_00385	Putative oxidoreductase ydbc	-1.54
GWH15_02245	1,4-beta-D-glucan glucohydrolase	-1.52
GWH15_11350	Putative KHG/KDPG aldolase	-1.51
GWH15_17080	Hypothetical protein	-1.49
GWH15_13390	L-rhamnonate dehydratase	-1.49
GWH15_01535	6-phosphogluconate dehydrogenase, NADP(+)-dependent	-1.49
GWH15_05340	General stress protein 69	-1.46
GWH15_13415	Xylitol oxidase	-1.46
GWH15_17570	D-galactarolactone cycloisomerase	-1.45
GWH15_07145	Long-chain-fatty-acid--coa ligase fadd15	-1.43
GWH15_16015	Putative propionyl-coa carboxylase beta chain 5	-1.42
GWH15_02240	Capsular glucan synthase	-1.42
GWH15_08525	Inosose dehydratase	-1.40
GWH15_01075	Catechol-2,3-dioxygenase	-1.36
GWH15_17815	Aldose 1-epimerase	-1.35
GWH15_14330	Scyllo-inositol 2-dehydrogenase (NAD(+))	-1.34
GWH15_18470	Adenine phosphoribosyltransferase	-1.32
GWH15_07615	4-hydroxy-4-methyl-2-oxoglutarate aldolase	-1.30
GWH15_02610	Monoterpene epsilon-lactone hydrolase	-1.26
GWH15_04030	Cystathionine beta-lyase metc	-1.23
GWH15_05255	Phosphoheptose isomerase	-1.23

GWH15_06910	4-alpha-glucanotransferase	-1.23
GWH15_11420	Copper-exporting P-type atpase A	-1.21
GWH15_13265	Hypothetical protein	-1.21
GWH15_14105	Free methionine-R-sulfoxide reductase	-1.20
GWH15_13500	Methylmalonate semialdehyde dehydrogenase [acylating]	-1.19
GWH15_11370	Gluconate 5-dehydrogenase	-1.17
GWH15_09530	Riboflavin biosynthesis protein ribba	-1.17
GWH15_18555	Ribonuclease R	-1.16
GWH15_06590	Hypothetical protein	-1.16
GWH15_14315	Hypothetical protein	-1.16
GWH15_12790	2-hydroxyhexa-2,4-dienoate hydratase	-1.14
GWH15_00640	Hypothetical protein	-1.14
GWH15_10795	Xylan 1,4-beta-xylosidase	-1.13
GWH15_02440	Acetyl-coenzyme A synthetase	-1.13
GWH15_11970	Aldehyde reductase yahk	-1.12
GWH15_06545	Putative glycerophosphoryl diester phosphodiesterase 1	-1.11
GWH15_02965	Hypothetical protein	-1.11
GWH15_13360	Ureidoglycolate lyase	-1.11
GWH15_14415	P-protein	-1.10
GWH15_11385	Hypothetical protein	-1.10
GWH15_05145	Hypothetical protein	-1.08
GWH15_15710	Putative glucose-6-phosphate 1-epimerase	-1.07
GWH15_06770	Hypothetical protein	-1.06
GWH15_14470	Hypothetical protein	-1.06
GWH15_14895	Oxalate decarboxylase oxdc	-1.03
GWH15_17295	Hypothetical protein	-1.03
GWH15_14850	Farnesyl diphosphate synthase	-1.03
GWH15_17980	Pyruvate dehydrogenase [ubiquinone]	-1.02
GWH15_08905	UDP-glucose 4-epimerase	-1.01
GWH15_10410	Trigger factor	-1.00
